# Computational Identification Raises a Riddle for Distribution of Putative NACHT NTPases in the Genome of Early Green Plants

**DOI:** 10.1371/journal.pone.0150634

**Published:** 2016-03-01

**Authors:** Preeti Arya, Vishal Acharya

**Affiliations:** 1 Functional Genomics and Complex System Lab, Biotechnology Division, CSIR-Institute of Himalayan Bioresource Technology, Council of Scientific and Industrial Research, Palampur- 176061, Himachal Pradesh, India; 2 Academy of Scientific and Innovative Research (AcSIR), CSIR-Institute of Himalayan Bioresource Technology (CSIR-IHBT) Campus, Palampur, Himachal Pradesh, India; University Hohenheim, GERMANY

## Abstract

NACHT NTPases and AP-ATPases belongs to STAND (signal transduction ATPases with numerous domain) P-loop NTPase class, which are known to be involved in defense signaling pathways and apoptosis regulation. The AP-ATPases (also known as NB-ARC) and NACHT NTPases are widely spread throughout all kingdoms of life except in plants, where only AP-ATPases have been extensively studied in the scenario of plant defense response against pathogen invasion and in hypersensitive response (HR). In the present study, we have employed a genome-wide survey (using stringent computational analysis) of 67 diverse organisms viz., archaebacteria, cyanobacteria, fungi, animalia and plantae to revisit the evolutionary history of these two STAND P-loop NTPases. This analysis divulged the presence of NACHT NTPases in the early green plants (green algae and the lycophyte) which had not been previously reported. These NACHT NTPases were known to be involved in diverse functional activities such as transcription regulation in addition to the defense signaling cascades depending on the domain association. In *Chalmydomonas reinhardtii*, a green algae, WD40 repeats found to be at the carboxyl-terminus of NACHT NTPases suggest probable role in apoptosis regulation. Moreover, the genome of *Selaginella moellendorffii*, an extant lycophyte, intriguingly shows the considerable number of both AP-ATPases and NACHT NTPases in contrast to a large repertoire of AP-ATPases in plants and emerge as an important node in the evolutionary tree of life. The large complement of AP-ATPases overtakes the function of NACHT NTPases and plausible reason behind the absence of the later in the plant lineages. The presence of NACHT NTPases in the early green plants and phyletic patterns results from this study raises a quandary for the distribution of this STAND P-loop NTPase with the apparent horizontal gene transfer from cyanobacteria.

## Introduction

About 470 to 500 million years ago (Ordovician period), the ancestors of modern flowering plants conquered and spread to almost of all the terrestrial habitat [1–4]; this time event led to the three major groups of land plants evolved: bryophytes (liverworts, hornworts and mosses), pteridophytes (lycophytes and monilophytes) and spermatophytes (seed-producing plants) with the latter dominating the nowadays habitats. Despite differences in the natural habitat and system complexity, a large number of similar features were found (as evidenced by similarities in pigmentation, cell-wall chemistry, biochemistry, and method of cell division) between early green plants (lycophyte, bryophyte and green algae) and modern land plants that suggested they shared common ancestor [5]. Besides, *Selaginella moellendorffii*, a spikemoss (having certain characteristic features that are also shared among land plants) is considered as a model organism for the comparative genomic studies [6] and considered as an important node in the evolution of plant life. The evolution is itself very interesting, associated with change, for the adaptation or survival of entities against the shifting environmental conditions. Therefore, with a rapid advancement in the next generation sequencing data, leading to reconstruction of the phylogenetic relationships among biological macromolecules, will be essential to identifying the innovations underlying the diversity of life.

The AP-ATPases and NACHT-NTPases are the members of signal transduction ATPases with numerous domain (STAND) P-loop NTPase class, play a crucial role in the signaling cascade of apoptosis and defense response against pathogens [7,8]. The STAND P-loop NTPase, a class of P-loop NTPases (phosphate-binding proteins), firstly defined by Leipe et al. [8], contain multiple domains including enzymatic (engaged in several signaling cascades), DNA or protein binding and superstructure-forming repeats (viz Toll-like Interleukin (TIR), Leucine Rich Repeat (LRR)) at carboxy-terminus or amino-terminus. The AP-ATPases are shared by animal apoptosis regulators Apaf-1 (human regulator of apoptosis), CED-4 (cell death protein 4 of *Ceanorhabditis elegans*) and plant disease resistance (R) proteins. These AP-ATPases are known to be involved in binding and hydrolysis of ATP as molecular function in defense response pathways and abbreviated as NB-ARC where NB stands for nucleotide binding; ARC for Apaf1, resistance gene (*R*) of plants and CED-4 [8,9]. In NACHT NTPases, the NACHT stands for NAIP (Neuronal apoptosis inhibitor protein), C2TA (MHC class 2 transcription activator), HET-E (Incompatibility locus protein from *Podospora anserina*) and TP1 (telomerase-associated protein) while the “NTPases” term used for nucleotide binding protein fold [10]. The NACHT NTPase can bind to either GTP or ATP, with preference for GTP over ATP [8]. In addition to defense response and apoptosis regulation, NACHT NTPases, are also engaged in the diverse functional activities such as transcription regulation in bacteria [8] and are widely spread in all kingdoms of life except plants.

The structure topology of STAND P-loop NTPase class was characterized by a central αβα fold containing regulatory units [11,12]. This fold contains two well-known conserved motifs: Walker A/P-loop/kinase-1a and Walker B/kinase-2 motif involved in the nucleotide-binding and hydrolysis. Both AP-ATPase and NACHT NTPase also contains this nucleotide-binding region that consists of several conserved motifs including Walker-A and Walker-B motif (ATPase/GTPase specific P-loop and Mg^++^-binding site respectively). However, the region of Walker-B motif was distinct in those two sister families that might be responsible for their differential biochemical functional activity [13].

The AP-ATPase (NB-ARC) is known to be present in diverse organisms ranging from bacteria to eukaryotes whereas NACHT NTPase is well reported in bacteria, fungi and animalia; however, it is absent in plants. In this study, for the first time, our stringent computational analysis has shown the presence of putative NACHT NTPases in early green plants: lycophyte (*Selaginella moellendorffii*) and green algae (*Chalmydomonas reinhardtii* and *Coccomyxa subellipsoidea*). With these observations, we get insight into the evolutionary history of both STAND P-loop NTPases involved in defense response and apoptosis regulation.

## Materials and Methods

### Sequence Retrieval

The whole protein sequence dataset of 40 different plant species, including 32 land plants and six green algae were retrieved from publicly available Phytozome v9.0 (http://www.phytozome.net/, last accessed: Feb, 2013; [14]). The protein sequences of six archaebacteria, three bacteria, three cyanobacteria, three protozoa, five fungi and seven species from animalia were downloaded from NCBI (http://www.ncbi.nlm.nih.gov/protein, last accessed: Feb, 2013). The Hidden Markov Model (HMM) profile of NACHT (PF05729.7) retrieved from Pfam [15] were used to screen the whole protein dataset for the identification of NACHT containing protein candidates in each organism by means of HMMER v3.1 (hmmsearch program) [16] with an e-value cutoff of 1e-04. A similar approach was carried out to find NB-ARC protein candidates in plants using HMM profile of NB-ARC domain (PF00931.17). The identified candidates were further confirmed for the presence of both NTPases (NACHT and NB-ARC) using PfamScan program with options -e_seq:1e-04; -e_dom:1e-04 and -clan_overlap where “-clan_overlap” option shows the information regarding overlapping domains belong to the same clan or family.

### Multiple Sequence Alignment (MSA) and Phylogenetic Analysis

The NACHT and NB-ARC NTPases specific amino acid sequences were extracted using in-house scripts and EMBOSS 6.6 (extractseq program) [17]. The extracted NACHT specific amino acid sequences were aligned using ClustalW v2.1 with default parameters [18]. The alignments were further employed for construction of the phylogenetic tree using RAxML version 7.2.8-ALPHA [19]. For finding the best empirical substitution model, we used ProtTest 3.4 [20] software package. The JTT +G+F was observed to be the best substitution model with respect to all four used statistical criteria—Akaike Information Criterion (AIC), Bayesian Information Criterion (BIC), Corrected Akaike Information Criterion (AICc) and Decision Theory (DT) Criterion [21]—with confidence interval of 100.0. The phylogeny was then inferred through Maximum likelihood (ML) statistical method with gamma model of rate heterogeneity and JTT matrix. GAMMA model parameters were estimated up to an accuracy of 0.1000000000 Log Likelihood units. The bootstrap analysis of 100 replicates was applied to evaluate the topological robustness of phylogenetic tree. MEGA v5.2.2 were used to visualize the phylogenetic tree [22].

In the case study of *S*. *moellendorffii*, MEGA v5.2.2 was used to evaluate the evolutionary patterns of NACHT and NB-ARC through neighbor joining method. The evolutionary distances were computed using the Poisson correction method. The topological robustness for each branch of phylogenetic tree generated was assessed by bootstrap analysis with 1000 replicates. Subsequently, a final consensus tree was constructed with a 50% cutoff. The multiple sequence alignment of NACHT and NB-ARC specific protein sequences were visualized using ClustalX v2.1 [23] and AliView v1.14 [24].

### Analysis of the conserved motifs

The protein sequences of NACHT and NB-ARC in *S*. *moellendorffii* were analyzed for the prediction of conserved potential patterns by employing the MEME V4.9.0 (Multiple Expectation Maximization for Motif Elicitation) [25]. The MEME analysis was done with a set of parameters i.e., minimum width: 8; maximum width: 20; maximum number of motifs: 20. The expected values and iterative cycles were set as default by MEME.

### *In silico* Structural comparison

The protein sequence of NACHT candidates of *S*. *moellendorffii* were used to build a model by means of I-TASSER web server which are based on threading approach [26]. The best model was selected on the basis of c-score (confidence score) calculated by I-TASSER to estimate the quality of predicted models. It is typically in the range of (-5,2), where a high value correlates with high confidence towards quality of model and vice-versa. The TM-score predicted by I-TASSER was also used for qualitative analysis of topology between two structure where a value of more than 0.5 infers the correct topology of predicted structure. TM-align program was implicated to compare protein structure [27] and PyMOL was employed for visualization of aligned structure [28].

## Results and Discussion

### Unusual presence of putative NACHT NTPases in early green plants

**Fig 1:**
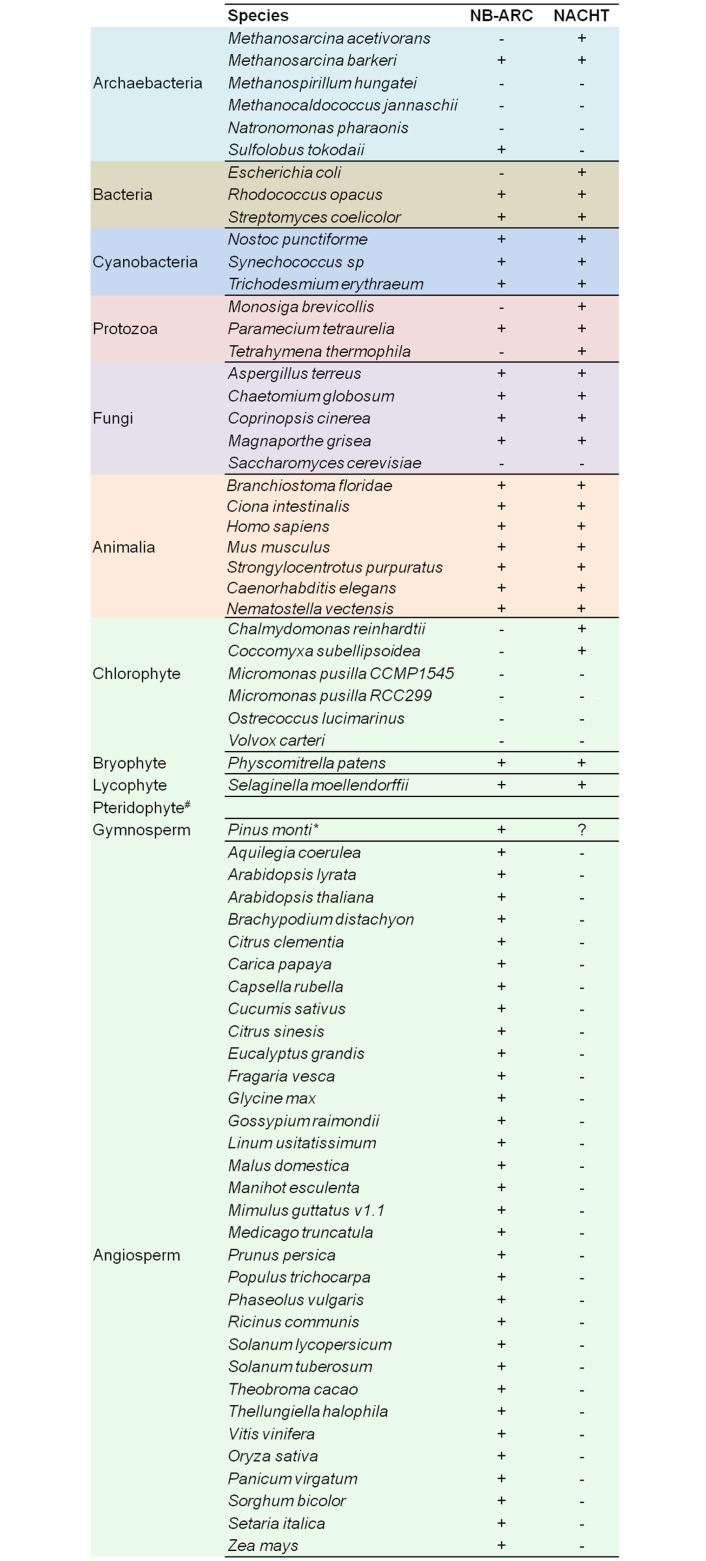
Genome-wide survey of 67 organisms for NB-ARC and NACHT NTPases. The genome-wide survey of 67 diverse organisms shows the presence (+) or absence (-) of NB-ARC and NACHT NTPases in respective organism.

**Fig 2:**
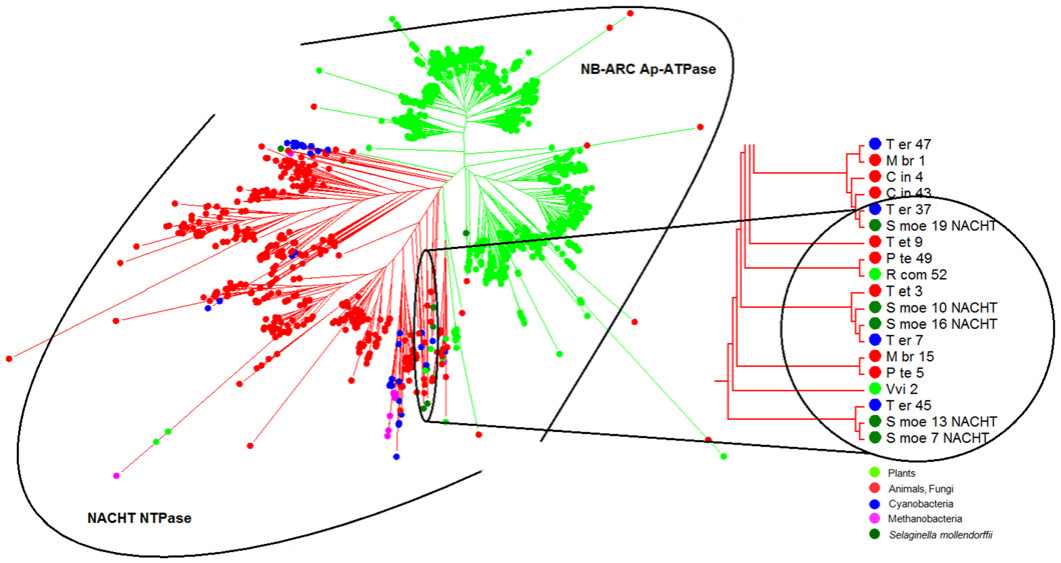
Phylogenetic tree of NB-ARC and NACHT NTPases of three major kingdoms of life. The NB-ARC and NACHT NTPases present in the diverse organisms were aligned by ClustalW v2.1 and phylogenetic tree was constructed by employing RAxML version 7.2.8-ALPHA. The phylogenetic tree was visualized using MEGA v5.2.2. Green and red branches denote the NB-ARC AP-ATPase and NACHT NTPase group respectively. The phylogenetic analysis inferred that few members of STAND P-loop NTPase of plants were making a clan in NACHT NTPase group, are putative NACHT NTPase of plants.

Recent genome-wide studies reported the presence of both types of STAND P-loop NTPases (NACHT and NB-ARC) in the diverse species. In this analysis, we have identified the presence of NACHT and NB-ARC NTPase in the genome of 67 organisms from diverse taxa (Fig 1; S1 Table). Fascinatingly, we found the presence of NACHT NTPase in the genome of green plants in addition to other eukaryotic species that was not reported previously and caught our attention for revisiting phylogenetic history. Therefore, to investigate the evolutionary relatedness of these NACHT NTPases, we performed multiple sequence alignment and reconstructed their phylogeny. From the phylogenetic analysis, it was observed that NACHT in plants grouped discretely (Fig 2); on the other hand, NACHT of archaebacteria, cyanobacteria, bacteria, protozoans, fungi and animalia were clustered separately where NACHT of few plant species were also making a clan which was beyond our expectation (Fig 2).

Later on analyzing the pfam results of NACHT specific sequences, we found that NACHT in most of the plants were overlapped with NB-ARC. However, the NACHT of *S*. *moellendorffii*, *C*. *reinhardtii*, and *C*. *subellipsoidea* were not observed to be overlapped with NB-ARC. Here, we used the term “NACHT NTPase” specific for NACHT to distinguish them from “NACHT overlap with NB-ARC”. Further sequence analysis of "NACHT overlap with NB-ARC" and few other STAND P-loop NTPase from other plant species form a clan within the NACHT NTPase group revealed the presence of NB-ARC specific sequences, were therefore considered as AP-ATPases (NB-ARC).

**Fig 3:**
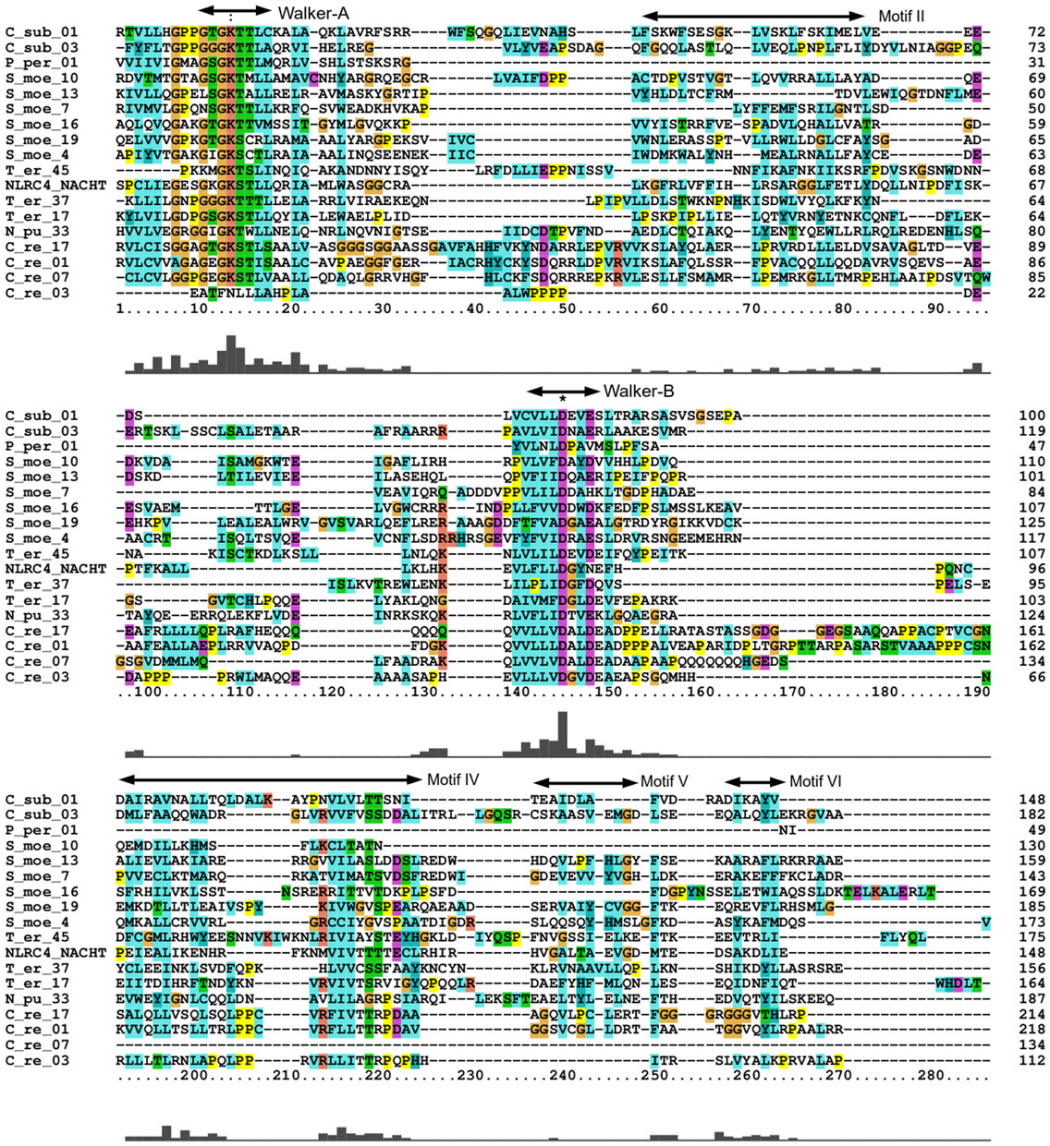
Multiple Sequence Alignment of NACHT NTPases. Multiple sequence alignment (MSA) for NACHT of *Selaginella moellendorffii*, *Physcomitrella patens*, green algae, cyanobacteria and NLRC4 showing the conserved region at the site of Walker-A and Walker-B and other motifs [10]. (The S_moe_* denotes NACHT NTPases in *S*. *moellendorffii*, C_re_* for *C*. *reinhairdtii* and C_sub_* for *C*. s*ubellipsoidea* where * represents any integer number). More than 80% consensus background coloring of amino acid residues reflects as follows: hydrophobic residues (ACFILMVWY), aromatic residues (FHWY), and aliphatic residues (VIL) are shaded cyan/blue; polar residues (STQN) are colored green; acidic residues (DE) are colored magenta; basic residues (KR) are colored red; Glycine (G) is shaded with mustard color; and Proline (P) is colored yellow.

In addition, one NACHT NTPase of short length but with well-conserved motif specific to this NTPase and significant e-value (4.3e-05) was found in *Physcomitrella patens*. The NACHT protein candidates were also identified in several green algal species (*C*. *reinhardtii* (4) and *C*. *subellipsoidea* (2)) (Table 1). To examine the NACHT NTPase at conserved amino acid residue level, we performed the MSAs with NACHTs from *S*. *moellendorffii*, *C*. *reinhardtii*, *C*. *subellipsoidea*, *P*. *patens*; NLRC4 NACHT (NP_001028539) were used as a NACHT representative. This analysis revealed the conservation of Walker-A and Walker-B motifs specific to NACHT NTPase (Fig 3). Whether so found NACHT is the sequence artifact, we also carried out blast search of all NACHT NTPases of the early green plants. The NACHT of *S*. *moellendorffii* were found mostly sharing a range of 15–20% sequence identity with well-known NACHT of human and mouse; green algae were found to share 21–35% similarity with NACHT of *Nostoc sp* PCC 7120 (S2 Table).

**Table 1:** List of NACHT domain containing protein sequences in early green plants.

Organism Name/ Accession Number	Name used in our study	Division	E-value	Length (Amino acids)
***Selaginella moellendorffii***		**Lycophyte**		
407161|PACid:15416689	S_moe_4		6e-05	172
417146|PACid:15401511	S_moe_7		1.2e-05	142
422201|PACid:15417115	S_moe_10		9.1e-07	129
424082|PACid:15421485	S_moe_13		1.1e-05	158
425148|PACid:15402815	S_moe_16		6.9e-05	168
444094|PACid:15413793	S_moe_19		4.1e-05	184
***Chalmydomonas reinhardtii***		**Green algae**		
Cre01.g039500.t1.2|PACid:27578790	C_re_01		5e-05	217
Cre03.g152550.t1.3|PACid:27576858*	C_re_03		1e-06	111
Cre07.g350451.t1.2|PACid:27564305	C_re_07		3.7e-05	133
Cre17.g723450.t1.3|PACid:27572133	C_re_17		2.7e-07	213
***Coccomyxa subellipsoidea***		**Green algae**		
14805|PACid:27390995	C_sub_01		6.6e-06	147
44528|PACid:27394369	C_sub_03		1.4e-06	181
***Physcomitrella patens***		**Bryophyte**		
Pp1s114_147V6.1|PACid:18037264*	P_per_01		4.3e-05	48

*Highly diverged /truncated putative NACHT NTPase

**Fig 4:**
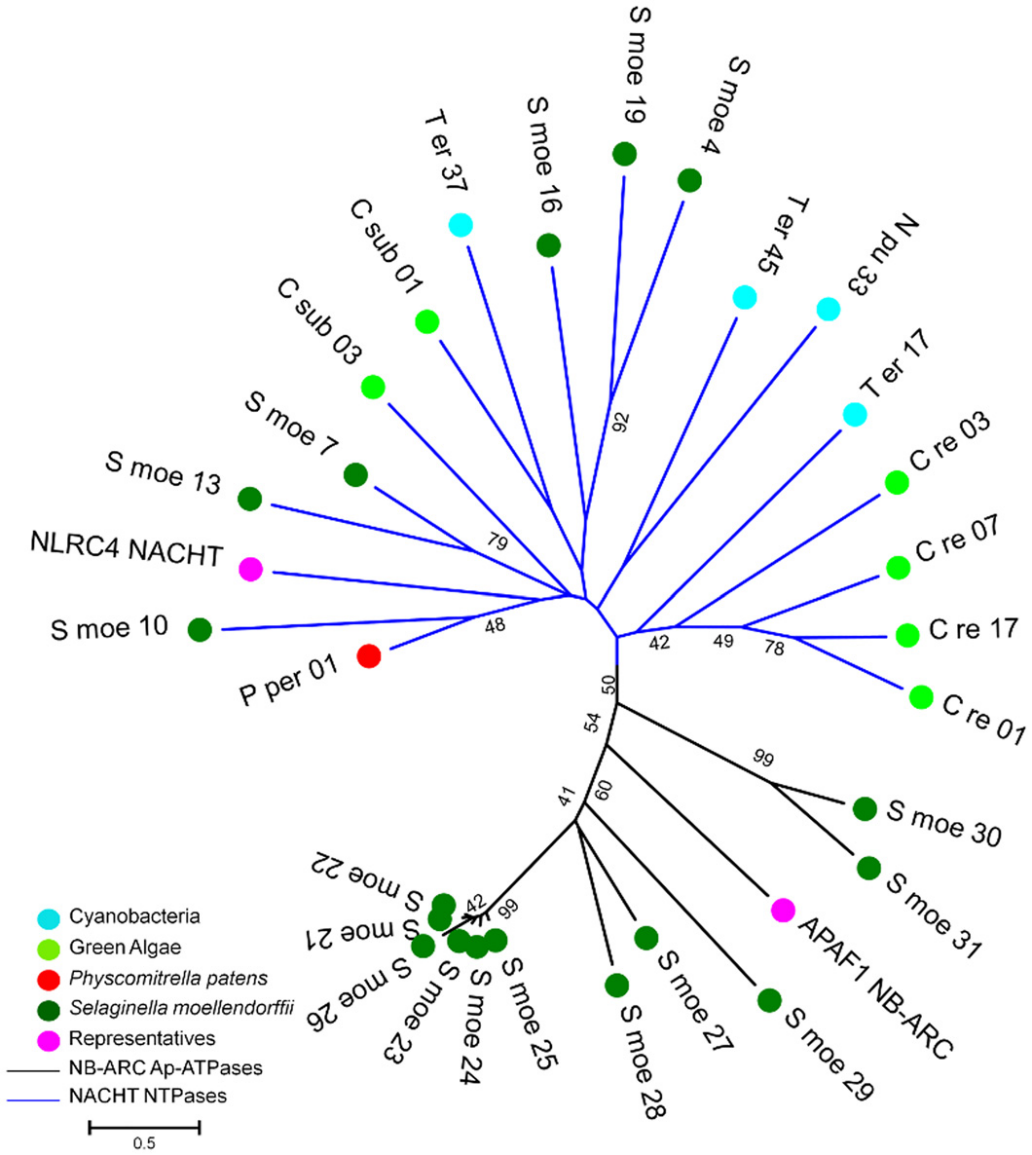
Unrooted phylogenetic tree of NB-ARC and NACHT NTPases. The members of NACHT and NB-ARC NTPases of *S*. *moellendorffii* were aligned with NACHT NTPases of green algae, cyanobacteria and *Physcomitrella patens* using ClustalW v2.1 and a phylogenetic tree was constructed by employing MEGA v5.2.2. The NLRC4 NACHT was taken as representative of NACHT and APAF1 NB-ARC as NB-ARC representative. (The S_moe_* denotes NACHT NTPases and NB-ARC AP-ATPases in *S*. *moellendorffii*, C_re_* for *C*. *reinhairdtii* and C_sub_* for *C*. s*ubellipsoidea* where * represents any integer number.)

To scrutinize the evolutionary fate of both STAND P-loop NTPases, identified NB-ARC and NACHT sequences were analyzed with molecular phylogenetic methods. While investigating this phylogeny, we have discarded some of the NB-ARC from our analysis if either of Walker-A or Walker-B motif were found to be totally absent. We used NLRC4 as a representative of NACHT (accession no: NP_001028539) and APAF1 (accession no: ABQ59028) as a NB-ARC representative for carrying out the phylogenetic analysis. The NACHT of cyanobacteria (that were grouped with *S*. *moellendorffii* NACHTs of NACHT NTPase group; Fig 2) were also included in this phylogenetic comparison. Interestingly, this evolutionary analysis suggests that both NACHT and NB-ARC arise as an independent entity during the evolution of STAND P-loop NTPases (Fig 4), further supporting their independent evolution [29].

**Fig 5:**
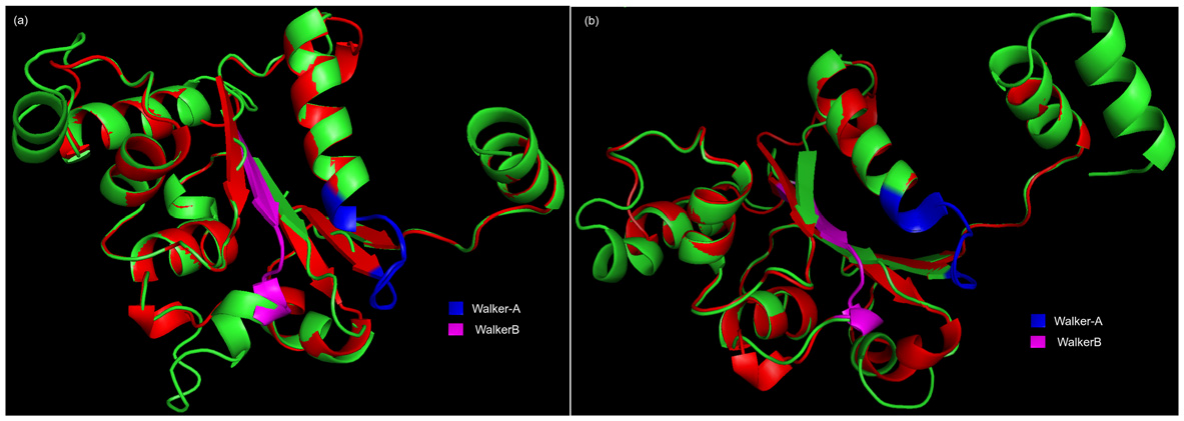
Structural comparison of NACHT of *Selaginella moellendorffii* with NLRC4 NACHT. The superposition of NACHT of *S*. *moellendorffii* with NLRC4 NACHT using TM-align. (a) NLRC4 NACHT and 407161|PACid:15416689 (b) NLRC4 NACHT and 425148|PACid:15402815. Red colour cartoon shows NLRC4 NACHT and green colour cartoons show *S*. *moellendorffii* NACHT domains. The residue range for Walker-A and Walker-B are 169–176 and 244–252 (NLRC4 NACHT); 82–89 and 169–177 (407161|PACid:15416689); 161–168 and 238–245 (425148|PACid:15402815) respectively. The quality of model was assessed on basis of c-score and TM-score. The predicted value of c-score and TM-score by I-TASSER are -1.36 and 0.55+-0.15 for 407161|PACid:15416689 and are -2.74 and 0.40+-0.13 for 425148|PACid:15402815. The root mean square deviation (RMSD) calculated by TM-align are 0.79 and 0.74 for 407161|PACid:15416689 and 425148|PACid:15402815 respectively.

To further evaluate NACHT at structural level, *in silico* fold recognition method was used to predict the structure for identified putative NACHT NTPase in *S*. *moellendorffii*. As expected, *in silico* predicted structure of *S*. *moellendorffii* NACHTs exhibit the same topological arrangements as that of experimentally derived structure of (PDB ID: 4KXF, chain K). The structure of NACHT comprises alternating alpha helix and beta sheets in the core region of Walker-A and Walker-B motifs. Interestingly, the structure of NLRC4 NACHT and *S*. *moellendorffii* NACHT superimpose well with a RMSD value of 0.79 Å (from NLRC4 NACHT and 407161|PACid:15416689) and 0.74 Å (from NLRC4 NACHT and 425148|PACid:15402815) (Fig 5) for Cα traces of the entire chain, despite relatively low sequence identity (< 30% sequence identity). To investigate the conserved residues responsible for functional activity, NACHT sequences of *S*. *moellendorffii* were fed to MEME. The consensus motifs in were found to be G[P/A]KG[S/T]GK[S/T] and hhhhD[RQG]AE respectively, which was strikingly similar to well-known NACHT of NLRC4. And as expected, in well-known NB-ARC architecture, Walker-A and Walker-B motifs of *S*. *moellendorffii*, similarly showed GA[GS]GAGKT and hhhhDDVW. Hence, one may presume the presence of another STAND P-loop NTPase as NACHT NTPase in this lycophyte plant. This inspection also inferred and supported that the divergence at Walker-B motif might have happened between these two NTPases which were responsible for the variation of biochemical activity: NTP binding and hydrolysis [13, 30].

The NACHT NTPase perform diverse function in addition to signal transduction in apoptosis because of different type of domain combinations (CARD-NACHT-WD40, PYD-NACHT-LRR, NACHT-WD40 or NACHT-LRR). However, we did not found any type of domain association in recently observed *S*. *moellendorffii* NACHT NTPases. Interestingly, we reported for the first time, the WD40 association at the C-terminus in the NACHT NTPases of chlorophyte, *C*. *reinhardtii*. The WD40 are the short motif of tryptophan and aspartic acid with 4–16 repeating units and involved in signaling pathways of programmed cell death (PCD) in eukaryotes [31, 32]. Furthermore, the delta-blast searches of NACHT NTPase of *C*. *reinhardtii* also showed the top similarity hits with a significant e-value for predicted NACHT NTPase (NACHT-WD40 protein) of *Nostoc* sp. PCC 7120 (sp|Q8YRI1.1|YY46_NOSS1) (S1 Table). The strikingly significant similarities of NACHT of *C*. *reinhardtii* with NACHT of *Nostoc sp* PCC 7120 may suggest their close evolutionary relationships among these STAND P-loop NTPases. The resulting observation is an indicative of the presence of NACHT NTPases in the early green plants which may be unusual regarding their unknown function in the plant genomes.

### Evolutionary scheme for horizontal gene transfer of NACHT NTPase genes in early green plants

**Fig 6:**
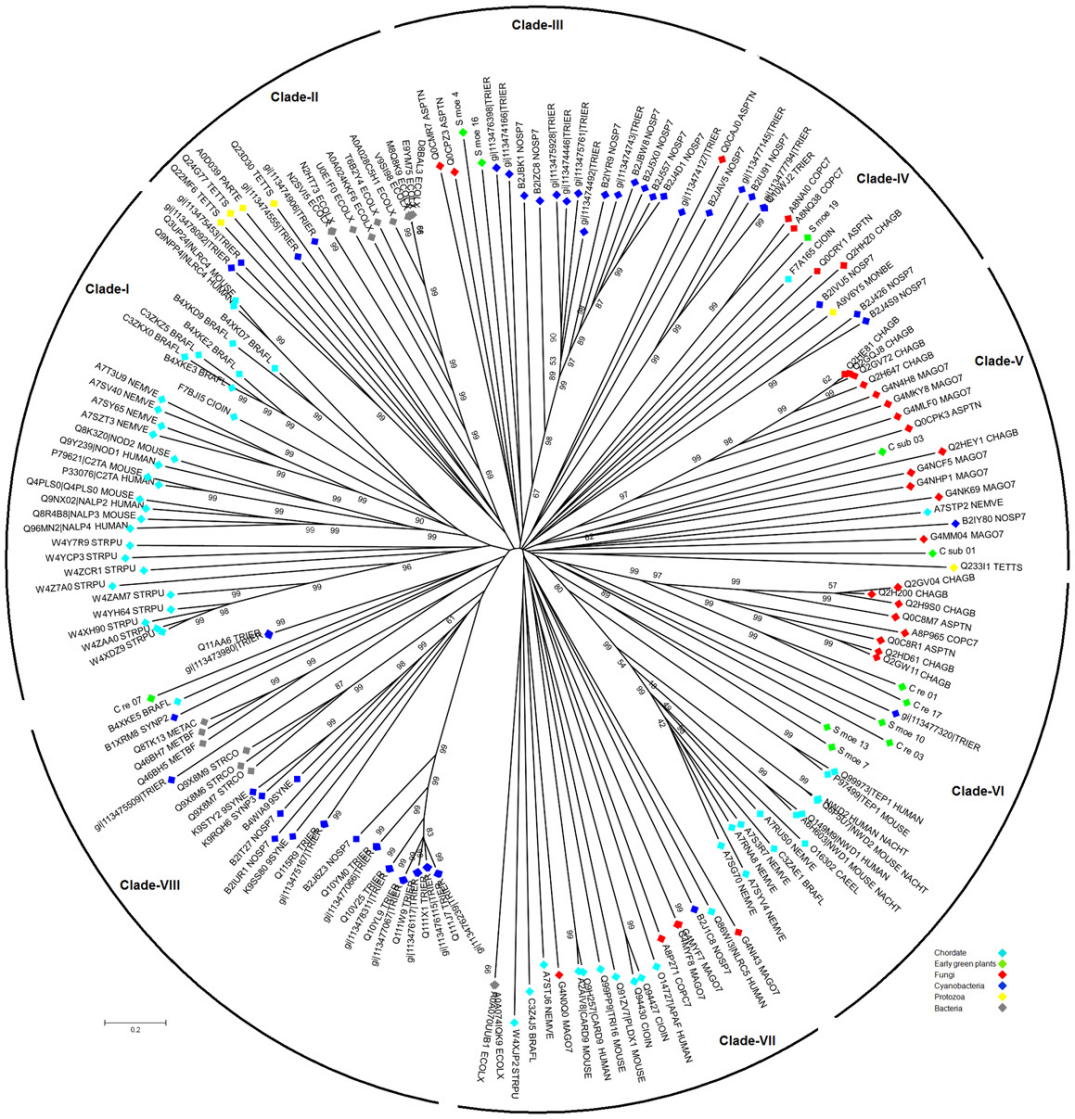
Unrooted phylogenetic tree of NACHT NTPases from diverse organisms of three major kingdoms of life. The multiple sequence alignment was performed for putative NACHT NTPases in early green plants (green algae, bryophyte, and lycophyte) and well-known NACHT NTPases in other organisms including bacteria, archaebacteria, cyanobacteria, protozoa, fungi, and chordates and phylogenetic tree was constructed using MEGA v5.2.2. The phylogenetic analysis shows that NACHT NTPases of early green plant were grouped in a clan of NACHT NTPases of cyanobacteria or fungi (in clades III, IV, V, VI, and III). Moreover, results of delta-blast search of NACHT NTPase in *C*. *reinhardtii* revealed the the top hits with *Nostoc* sp PCC 7120. From phyletic patterns and delta-blast search results from the present study, we infers that the DNA for NACHT in green algae may have been transferred from the ancestors of extant cyanobacteria.

**Fig 7:**
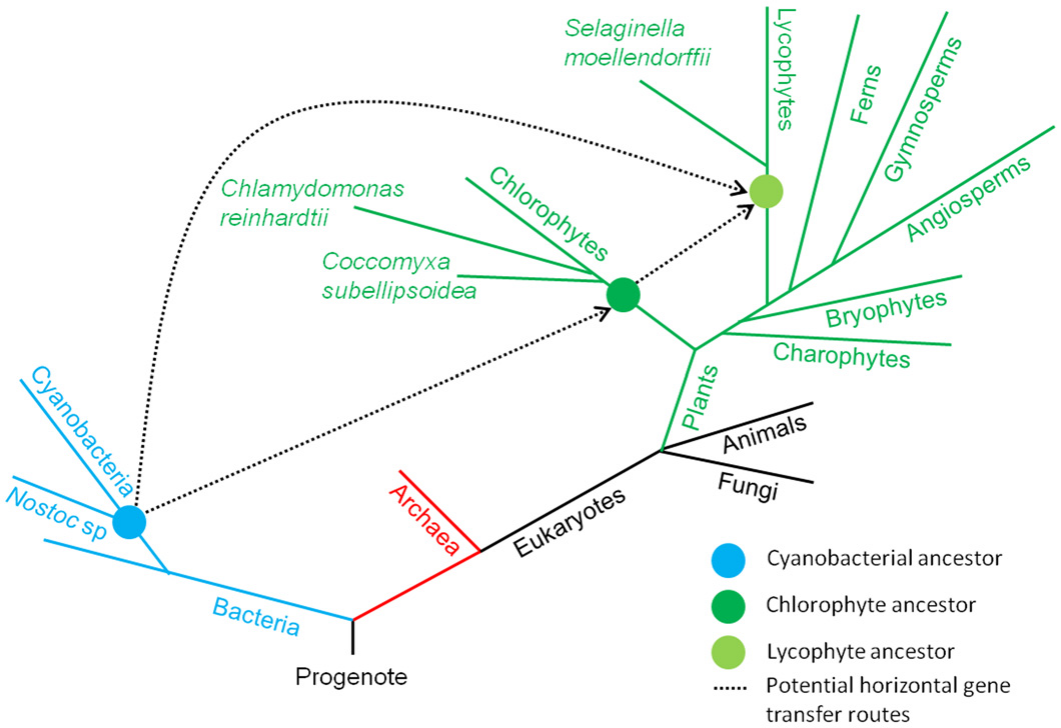
Hypothetical pictorial representation of horizontal gene transfer of NACHT NTPase genes in early green plants. Probabe horizontal gene transfer (HGT) of NACHT NTPase from cyanobacterial ancestor to early green plant lineages. The NACHT NTPase occurs in all three major kingdoms of life that arose from single progenote and may have acquired in single-celled green alage (chlorophytes) through HGT from an ancestor of cyanobacteria (*Nostoc* sp.).

The Ordovician period was heralded with biggest mass extinction lead to the loss of life. At that time period, the green algae was likely to be dominated flora and then first terrestrial plant life have been evolved. The NACHT NTPases were involved in signaling cascades of apoptosis/programmed cell death (PCD) which compliment the cell development and differentiation in the diverse organisms of three major kingdoms of life. These NACHT NTPases has been later lost in plant lineages however still present in the genome of early green plants (*S*. *moellendorffii*, *C*. *reinhardtii* and *C*. *subellipsoidea*) raises a quandary for their distribution. To inspect the phenomenon behind the acquisition of NACHT NTPases in early green plants, we performed the phylogenetic comparison of identified NACHT NTPases in the present study and some well-known NACHT NTPases from different organisms. The NACHT of early green plants were grouped either with NACHT of cyanobacteria or fungi in clades III, IV, V, VI, and VIII (Fig 6; S3 Table). However, the delta-blast results revealed the top hits with NACHT NTPase of *Nostoc sp* for NACHT in *C*. *reinhardtii*. The clan of NACHT of green algae, *C*. *reinhardtii* grouped with cyanobacterial NACHT and further the blast search result showing the highest similarity with *Nostoc* sp PCC 7120 infers that the DNA for NACHT in green algae may have been transferred from the ancestors of extant cyanobacteria. The transmission of genetic information in progeny from parents usually occurs by vertical gene transfer (VGT) by means of sexual and asexual reproduction in the complex multicellular eukaryotes. However, recent reports assessed the importance of horizontal gene transfer (HGT) in the evolution of eukaryotic genomes which was considered to be predominant in prokaryotes [33, 34]. HGT, often known as lateral gene transfer (LGT), is the process of introducing the novel genes between evolutionary unrelated species [35], thus facilitating phenotypic variation and adaptation to the changing environments. Incongruent relationships observed in the molecular phylogenetic trees is the general method used to detect the HGT events in unrelated species [36]. During the evolution, plants gradually developed complexity in their biological systems to survive themselves to the shifting environments where HGT also played a significant role in this adaption [37]. Previous reports have also documented that cyanobacterial invasion and subsequent HGT is the likely resource for the evolution of diverse types of proteins in the modern flowering plants [38–41]. Moreover, HGT served as a frequent scenario for integration of microorganismal genes into the plant nuclear genomes, speculated in a few phylogenetic studies of thousands protein-encoding genes [42–46]. In addition, previous studies also divulged the cyanobacteria as a well-known symbioints and cyanobioints for wide spectrum of fungi and plants (bryophytes, petridophytes, and spermatophytes [47]. Based on the current insights on phylogeny of NACHT NTPases, close homology relationship of NACHT NTPases of *C*. *reinhardtii* with NACHT NTPases of *Nostoc sp*. (S2 Table) and absence in other plant species suggested that NACHT NTPase might have been acquired in early green plant lineages through HGT events (Fig 7). The plausible reason for the absence of NACHT NTPases in other plant species is that their function might have been overtaken by a large repertoire of NB-ARC ATPases. The observation of putative NACHT NTPases distribution in early green plants adds a probable item to the list of functionalities acquired through HGT in green plants from the ancestors of extant cyanobacteria. However, further investigation will be needed regarding current lack of data to assess the HGT scenario of NACHT NTPases in early green plants with greater clarity.

### *Selaginella moellendorffii* as an important evolutionary node

**Fig 8:**
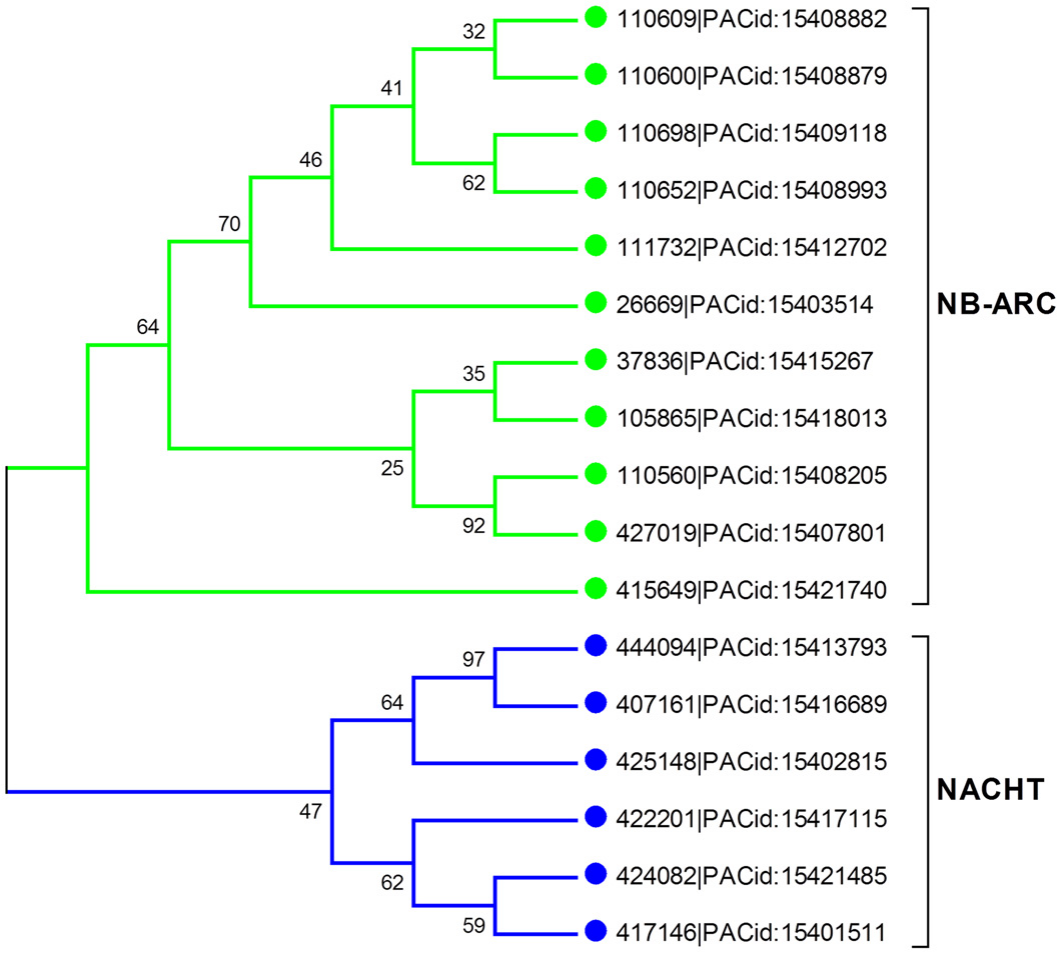
Phylogenetic tree of NB-ARC and NACHT NTPases in *Selaginella moellendorffii*. The NACHT and NB-ARC of *S*. *moellendorffii* were aligned using ClustalW and phylogenetic tree was constructed using MEGA v5.2.2. The green lines represents the NB-ARC AP-ATPases and blue lines denotes NACHT NTPases proteins forming two separate clades. The members of both NACHT and NB-ARC NTPase grouped discretely supporting the independent evolution of these STAND P-loop NTPases.

*S*. *moellendorffii* is the member of the oldest living vascular plant lineage which lack true leaves and roots. It first appeared in fossil record 400 million years ago and considered as a model organism for comparative genomic studies [6]. The phylogenetic analysis of the identified AP-ATPases (NB-ARC) and NACHT-NTPases in this lycophyte divulges the two discrete clades specific for NB-ARC and NACHT respectively (Figs 4 and 8), suggesting the independent evolution supported by previous literature [29]. The sequence, motif analysis and structural considerations confirm the presence of NACHT in addition to NB-ARC NTPases in this ancient vascular plant. Moreover, the complement of NB-ARC and NACHT was found to be in commensurable number in comparison to the large repertoire of NB-ARC in other land plant species that make *S*. *moellendorffii* more interesting for studying the evolutionary tree of life.

Few reports documented more similarity for the organelle genome composition in *S*. *moellendorffii* with chlorophyceae in comparison with other land plants [5] whereas other studies also demonstrated the close relationship for the identified secondary metabolites in *S*. *moellendorffii*, a non-seed plant, with a highly advanced flowering plants [48]. In the case of *S*. *moellendorffii*, both NB-ARC and NACHT were found to be in comparable number (11 NB-ARC and 6 NACHT) whereas other land plants show a large repository for NB-ARC; for example, the bryophyte, *P*. *patens* shows a higher number of NB-ARC (88). This observation further led us to exemplify the significance of this extant lycophyte in the evolution of STAND P-loop NTPases and suggests *S*. *moellendorffii* as an important piece of the puzzle in understanding the evolution of land plants [6].

Our report of NACHT NTPase acquisition in early green plants raises a question: why it had never been pervaded in other green plants? We hypothesize that the absence of associated domains responsible for NACHT function is the most likely reason for the loss of NACHT NTPase in plants. Moreover, the large complement of NB-ARC in plants may overtake the function of NACHT, which is the another possible explanation behind the absence of NACHT NTPase in green plant species. The evolutionary analysis revealed that cyanobacteria may have acted as donors of genetic materials for more than thousand proteins in the modern flowering plants as result of HGT or endosymbiotic gene transfer [38–41]. Our finding of the close homology between NACHT NTPase in *C*. *reinhardtii* and *Nostoc sp* PCC 7120 proteins demonstrates that HGT may have played a decisive role in the distribution of the NACHT domain in the early green plants from the free-living cyanobacterial ancestor species. Most interestingly, the comparable number of both STAND P-loop NTPases, in the extant lycophyte, *S*. *moellendorffii*, supports their independent origin. This study states the independent origin of both STAND P-loop NTPases in eukaryotes and invasion of NACHT NTPases in early green plants with apparent HGT.

## Supporting Information

S1 Table List of protein sequence accession number used for phylogenetic analyses in the report from different species. (XLS)

S2 Table Blast hits of corresponding protein sequences of early green plant lineages. (XLS)

S3 Table List of NACHT NTPases used for phylogenetic analysis in Fig 6. (XLS)
